# Nursing Affairs in Ireland

**Published:** 1917-08-18

**Authors:** 


### THE EDITOR'S LETTER-BOX.

### Nursing Affairs in Ireland.

*To the Editor of* The Hospital.

The Irish Probationer's Unhappy Position.

Sir,—Touching your remarks in last issue dealing with the need for the reform and reorganisation of the nursing profession in Ireland I do not presume to dogmatise, but I venture to suggest for your consideration that betterment, if it is to be general, must begin with the probationer. Her remuneration and conditions of service regulate the entire salary scale, and in Ireland, speaking generally—for there are one or two exceptions—the probationer is regarded not as a necessary expense, but as a legitimate and almost indispensable source of revenue. She is an involuntary contributor to the hospital funds. This condition of affairs is due in great measure to the difficulty and expense with which a candidate probationer for employment in Great Britain is confronted. Naturally and properly no matron cares to accept a probationer without a personal interview, nor will a matron care to share any risk that a candidate may run in undertaking a long journey in order to be interviewed. Hence it becomes necessary for an Irish girl seeking employment as probationer in an English hospital to travel thither and submit references which must seem very far off, and difficult, if not impossible, for the hospital authorities satisfactorily to verify. The problem is a serious one, and what happens in a very large number of cases is that the probationer elects to find the entrance fee to a local hospital and to work through her three years of probationership for a nominal reward.

Seeing that this system prejudicially affects the entire profession, the problem ought to be boldly faced, but it must be admitted that so long as the supply of passable candidates possessing the necessary financial qualification is adequate a remedy will be difficult to find. The obvious reply silences criticism—" Our list of applications far exceeds our demand. Why offer better terms? "

A Healthy and Growing Branch of the College.

Now that we have in our midst a healthy and growing Branch of the College of Nursing, one of whose most attractive attributes is its connection with Great Britain, would it not be possible to form a responsible Selection Committee whose function would be to provide suitable candidates for hospitals in Great Britain? This, if established, would save the would-be probationer the risk and expense of a possibly fruitless journey to England, and at the same time would tap the supply at its source. We have at present Selection Committees for all kinds of employments in connection with war work, and they achieve most satisfactory results. I venture to suggest that a practical task of this kind undertaken by the Irish Branch of the College would have far-reaching effects in other directions than that indicated here, which in itself is of the highest importance.

The Midwives Bill for Ireland.

Public opinion at the moment ought to be strongly directed towards the Midwives Bill which is at present in draft form in the offices of the Local Government Board, Dublin. It has been for long in this form, and is likely so to remain unless pushed forward by forces which command the attention of the Government. One of its strongest and most earnest advocates here is Sir Andrew Horne, a member of the Irish Board of the College of Nursing. It is difficult to understand what can have blinded the eyes of the Irish Nurses' Association to the desirabilty of obtaining a Midwives Act when the English Bill was passing through Parliament. Doubtless those who offered opposition then have long since bitterly repented. Since Scotland obtained an Act of Parliament, Ireland has become more then ever the dumping ground of the "handy woman," who has been put out of business in Great Britain. The same blindness still obtains in connection with the College of Nursing, and the most curious phase of the phenomenon is that the leaders of the opposition are neither Irish women nor Irish trained. So far as the Midwives Bill is concerned, Mr. Duke has promised if possible to introduce it in Parliament, provided that it is non-contentious. Is it possible to produce a nursing measure that is non-contentious, even in war-time, even though infant mortality in Ireland beats the record, and qualified midwives are unobtainable? We shall see.—Yours very truly. IERNE.

